# Scintillation Fiber-Optic Detectors for Dosimetry of 60 MeV Proton Beam

**DOI:** 10.3390/ma19173724

**Published:** 2026-09-01

**Authors:** Sandra Witkiewicz-Lukaszek, Bogna Sobiech, Paweł Bilski, Anna Mrozik, Michał Sądel, Jan Swakoń, Damian Wróbel, Janusz Winiecki, Mark Akselrod, Yuriy Zorenko

**Affiliations:** 1 Faculty of Physics, Kazimierz Wielki University in Bydgoszcz, Powstańców Wielkopolskich Street 2, 85-090 Bydgoszcz, Poland; sanwit@ukw.edu.pl; 2 The Franciszek Łukaszyk Oncology Center, Medical Physics Department, Dr. Izabeli Romanowskiej Street 2, 85-796 Bydgoszcz, Poland; bognasobiech@gmail.com (B.S.); janusz@co.bydgoszcz.pl (J.W.); 3 Institute of Nuclear Physics, Polish Academy of Sciences, Radzikowski Street 152, 31-342 Krakow, Polandmichal.sadel@ifj.edu.pl (M.S.); jan.swakon@ifj.edu.pl (J.S.); damian.wrobel@ifj.edu.pl (D.W.); 4 Department of Oncology and Brachytherapy, Collegium Medicum in Bydgoszcz Nicholas Copernicus University in Toruń, Jagiellońska Street 13/15, 85-067 Bydgoszcz, Poland; 5 Physics Department, Oklahoma State University, 145 Physical Sciences Building, Stillwater, OK 74078, USA; akselrod.mark@gmail.com

**Keywords:** proton therapy, scintillation detectors, fiber-optic dosimetry, real-time beam monitoring, radioluminescence, GAGG:Ce, Al_2_O_3_:C, Al_2_O_3_:C,Mg

## Abstract

Scintillation fiber-optic detectors (FODs) offer a compact, electrically passive, and versatile solution for real-time proton beam monitoring in clinical radiation therapy. In this work, we systematically investigate and compare FODs based on GAGG:Ce, Al_2_O_3_:C, and Al_2_O_3_:C,Mg crystal scintillators under identical proton irradiation conditions. The detectors were evaluated using a 60 MeV clinical proton beam over a dose range of 0.5–15 Gy, with radioluminescence spectra acquired at integration times as short as 70 ms. The GAGG:Ce-based FOD exhibited the highest signal intensity, a fast temporal response, and excellent stability under repeated irradiation cycles, enabling reliable real-time dose monitoring. In contrast, the FODs based on Al_2_O_3_:C and Al_2_O_3_:C,Mg demonstrated high sensitivity, reproducible dose–response characteristics, and a low effective atomic number (Z_eff_ ≈ 11), providing near tissue-equivalent behavior. Mg co-doping modified the luminescence characteristics of Al_2_O_3_:C by enhancing the contribution of visible emission centers. However, under the present proton irradiation conditions, the overall RL signal remained lower than that of Al_2_O_3_:C while preserving good signal stability. All detector configurations maintained consistent performance across multiple irradiation cycles, with GAGG:Ce showing the lowest signal variation. Overall, these results demonstrate that scintillation FODs constitute a robust platform for proton beam diagnostics and dosimetry. GAGG:Ce-based detectors are shown to have promising potential for real-time proton beam monitoring, whereas sapphire-based detectors provide advantages for dosimetric applications where tissue equivalence is essential.

## 1. Introduction

Proton beam therapy has become one of the most advanced modalities in modern radiation oncology due to its superior dose conformity and the presence of a well-defined Bragg peak, which allows for maximal energy deposition within the tumor volume while sparing surrounding healthy tissues [1,2]. However, the clinical advantages of proton therapy strongly depend on precise beam delivery and accurate dosimetric verification. Consequently, the development of reliable, real-time proton beam monitoring systems remains a critical challenge in both clinical and research environments [3].

Conventional proton beam dosimetry relies on ionization chambers, semiconductor detectors, radiochromic films, and thermoluminescent (TL) dosimeters [4,5,6]. While these techniques provide high accuracy, they often suffer from intrinsic limitations such as spatially extensive detector configurations, sensitivity to electromagnetic interference, limited temporal resolution, or the inability to provide real-time feedback during irradiation [7]. In addition, some detector types introduce perturbations to the proton beam or require complex calibration and correction procedures [8] (see Table S1 in the Supplemental Material).

Scintillation fiber-optic detectors (FODs) have emerged as a promising option for proton beam dosimetry and monitoring [9,10,11]. Their operating principle is based on the conversion of ionizing radiation into visible or near-visible photons within a scintillator, followed by optical signal transmission through a flexible fiber to a remote photodetection system. This configuration provides several key advantages, including compact detector size, electrical insulation, immunity to electromagnetic interference, and the possibility of positioning sensitive electronics outside the radiation field [12].

The compact FOD sensor heads, incorporating scintillation crystals sized 1–2 mm × 1–2 mm × 0.5–1 mm, are well suited for radiation proton beam applications. Their compact dimensions enable straightforward integration into standard medical tools, such as urinary catheters, for real-time dose monitoring. The measured dose can then be compared with the planned dose distribution to confirm that the patient receives the prescribed treatment.

The distinct advantages of such systems include real-time dosimetry, compact size, and high spatial resolution. Integration into the measurement setup or treatment environment can be achieved through various approaches, such as coating the fiber with a scintillating material, coupling it to a photodetector, or embedding the scintillator within the fiber structure.

An important advantage of scintillation FODs is their capability for real-time beam monitoring with high temporal resolution, which is particularly relevant for modern proton therapy techniques such as pencil beam scanning and intensity-modulated proton therapy [13,14]. Moreover, the use of different scintillation materials enables tailoring of detector properties such as sensitivity, radiation hardness, and response linearity [15].

To our knowledge, this work presents the first systematic comparison of scintillation FODs based on Gd_3_Al_2_Ga_3_O_12_:Ce garnet crystals as well as Al_2_O_3_:C and Al_2_O_3_:C,Mg sapphire crystals, which was performed under identical clinical proton beam conditions.

## 2. The Choice of Scintillation Material for FODs

Despite increasing interest in scintillation-based proton detectors, systematic comparative studies of different scintillator materials integrated into fiber-optic systems remain limited, particularly under identical experimental conditions and clinically relevant dose ranges [16]. In particular, there is a need to evaluate detector sensitivity, dose linearity, temporal response, and long-term stability under repeated proton irradiation.

The choice of scintillation material plays a decisive role in FOD performance under proton irradiation. High-density, high-Z scintillators such as GAGG:Ce garnet provide strong radioluminescence (RL) signals and fast decay times, making them well suited for applications requiring high signal-to-noise ratios and rapid temporal responses [17,18,19] (Table 1). In contrast, low-Z materials such as Al_2_O_3_:C and Al_2_O_3_:C,Mg sapphire exhibit dose responses closer to water and human tissue, which is advantageous for accurate dosimetry in clinical conditions [20,21,22,23,24]. Co-doping with magnesium has been shown to modify trapping and recombination mechanisms, leading to enhanced luminescence efficiency and improved detector sensitivity [23,24].

The first material for scintillation heads in the present work was a piece of a commercially available, highly efficient scintillation crystal of Gd_3_Al_2_Ga_3_O_12_ (GAGG:Ce) garnet, produced using the Czochralski method at 1850 °C in an Ar + 1.5% O_2_ atmosphere by C&A Ltd., Sendai, Japan [18]. The second material selected for investigation in this work was pieces of commercially available, well-known dosimetric crystals of carbon-doped and carbon–magnesium co-doped Al_2_O_3_ (sapphire), grown using the Czochralski (Cz) method in the Stillwater Crystal Growth Division of Landauer Inc., Glenwood, IL, USA, under a highly reducing atmosphere in the presence of graphite [20,21]. The concentration of dopants in the crystals were 0.1 at.% (C) and 0.2 at.% (Mg) in the sapphire and 0.21 at.% (Ce) in the garnet.

The luminescence properties of the studied scintillation materials were investigated by cathodoluminescence (CL) and radioluminescence (RL) spectroscopy at room temperature. CL spectra were measured using a JEOL JSM-820 scanning electron microscope (JEOL, Tokyo, Japan) operated with an electron beam energy of 20 kV. The emitted radiation was collected and analyzed with a StellarNet grating spectrometer equipped with a CCD detector (StellarNet, Inc., Tampa, FL, USA), providing a spectral coverage from 200 to 950 nm.

The RL spectra generated by the crystal detectors were recorded using an AvaSpec-HERO spectrometer (Avantes, Nynomic AG, Apeldoorn, The Netherlands). For the sapphire-based crystals, the spectral range was 320–630 nm, whereas for the garnet crystal, it extended from 320 to 800 nm. Spectra were acquired both with a 70 ms (only for GAGG:Ce) and 700 ms (for all scintillators) integration time throughout the entire irradiation period, corresponding to doses between 0.5 and 15 Gy. The absence of signal below 320 nm results from the UV cutoff of the spectrometer’s glass optics.

**Figure 1:**
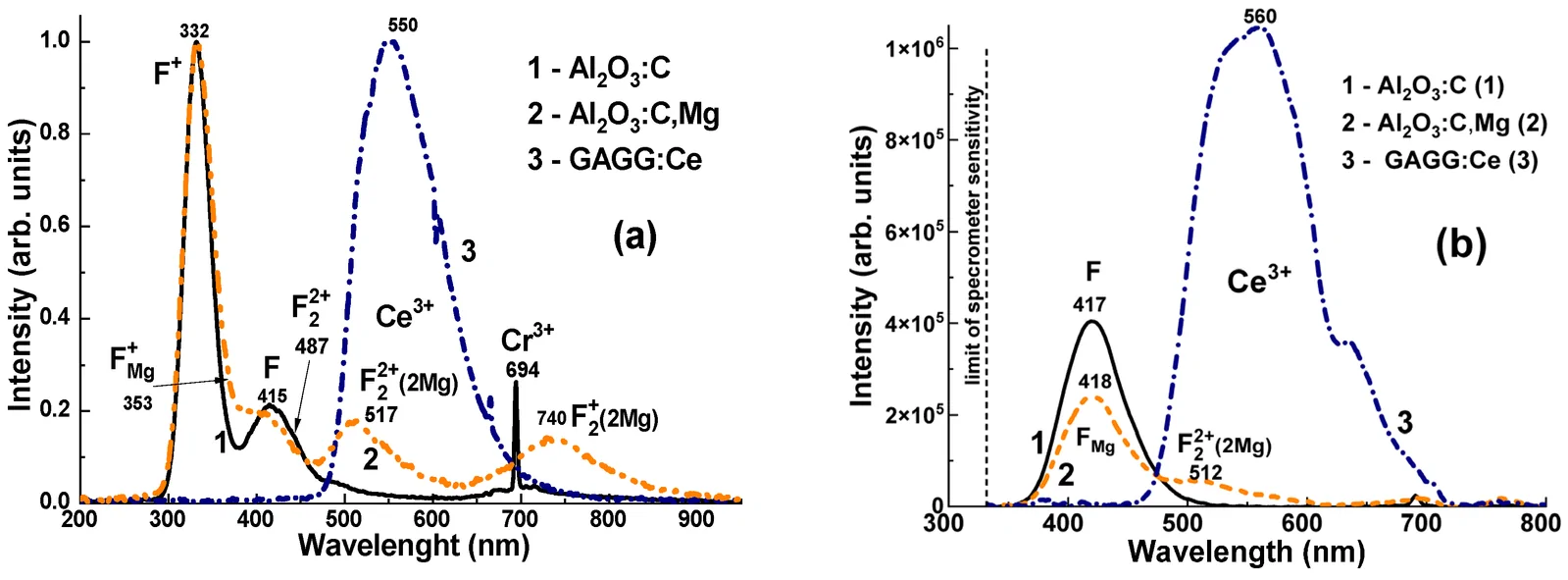
Normalized CL spectra (**a**) and RL (**b**) spectra of Al_2_O_3_:C (1), Al_2_O_3_:C,Mg (2) and GAGG:Ce (3) crystal detectors, recorded under e-beam excitation (**a**) and γ-ray excitation from ^192^Ir source using developed FODs with the different scintillation-based heads (see also [28]). T = 300 K.

The CL and RL spectra of the Al_2_O_3_:C, Al_2_O_3_:C,Mg, and GAGG:Ce crystal detectors are shown in Figure 1. The emission spectra of the Al_2_O_3_:C crystal (Figure 1a,b, curves 1) consist of a superposition of the dominant F^+^ and F center luminescence bands, which peak at 332 and 415 nm, respectively, along with low-intensity bands at 487 and 707 nm, attributed to F_2_^2+^ and F_2_^+^ pair centers [20,21]. Doping the sapphire host with Mg^2+^ significantly modifies the electronic structure of F-like emission centers in the Al_2_O_3_:C,Mg crystal [23]. The emission spectra (Figure 1a,b, curves 2) show dominant F_Mg_^+^ (333 nm) and F_Mg_ (≈415 nm) emission bands, together with relatively intense visible bands at 517 and 740 nm, corresponding to F_2_^2+^(2Mg) and F_2_^+^(2Mg) aggregate centers, respectively [23].

The F_Mg_^+^ and F_Mg_ emission bands in the 400–450 nm range strongly overlap with the absorption/excitation band of the F_2_^2+^(2Mg) centers at 435 nm [22]. In addition, the green emission band at 517 nm partially overlaps with the excitation band of the F_2_^+^(2Mg) centers at 620 nm [23]. Consequently, luminescence from F_Mg_^+^ and F_Mg_ centers can excite emission from F_2_^2+^(2Mg) and F_2_^+^(2Mg) centers in the Al_2_O_3_:C,Mg crystal [23]. Moreover, the F^+^, F, F_2_^2+^, and F_2_^+^ centers in Al_2_O_3_:C, as well as the F_Mg_^+^, F_Mg_, F_2_^2+^(2Mg), and F_2_^+^(2Mg) centers in Al_2_O_3_:C,Mg, act as efficient trapping centers [20,21,22,23,24]. As a result, energy transfer from the sapphire host to oxygen-defect-related UV and visible emission centers may be delayed when these crystals are used as scintillators.

The emission spectrum of the GAGG:Ce crystal (Figure 1a,b, curves 3) shows strong yellow–green luminescence with a peak at 550–560 nm, corresponding to the 5d^1^–4f transitions of Ce^3+^ ions in the garnet matrix [18,19,25]. Notably, GAGG:Ce exhibits minimal UV emission from antisite defect (AD)-related centers, such as Gd_Ga_ and Gd_Al_ [24]. This behavior is attributed to compositional band-gap engineering [18,19,25] and to depopulation (“burning”) of the AD energy levels via lower conduction-band states formed mainly by the Ga^3+^ 3d levels [26]. As a result, GAGG:Ce offers a clear advantage over Al_2_O_3_-based crystals, particularly in terms of RL intensity stability with irradiation time (dose) [27,28,29].

## 3. Experimental Equipment and Results

### 3.1. Proton Beam

**Figure 2:**
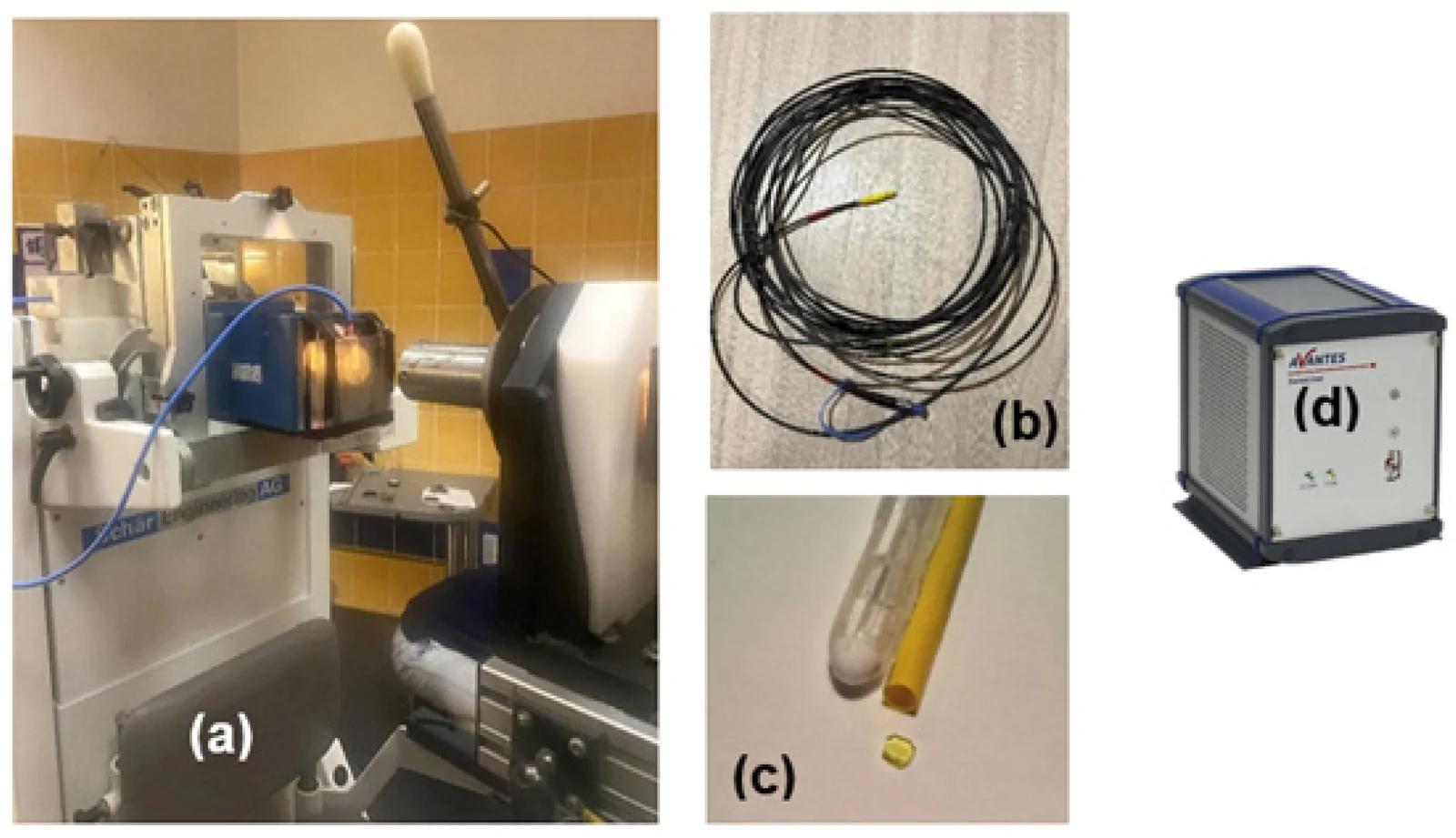
Proton beam irradiation facility at IFJ PAN, Kraków, Poland (**a**), the 7.7 m long optical fiber (**b**) with scintillation head and the base of GAGG:Ce crystal (**c**), and Avantes-HERO spectrometer for RL spectra measurements (**d**).

Exposure of the FODs to a proton beam was performed at a horizontal small-field proton beam irradiation facility (built and formerly used for proton eye radiotherapy) [30,31,32] using a 60 MeV proton beam produced by an AIC-144 isochronous cyclotron at IFJ PAN, Kraków, Poland (Figure 2a).

During irradiation, the tested detectors were positioned within a passively scattered fully modulated SOBP (Spread-out Bragg Peak), in a range of 29 mm in water at a 14 mm water equivalent depth. The detectors were placed in the dedicated polymethyl methacrylate (PMMA) phantom, within a uniformly collimated radiation field with a diameter of 40 mm. Dosimetry calibration of the irradiation station was performed in accordance with the recommendations of IAEA TRS 398 protocol (IAEA TRS-398) [4] using a Semiflex ionization chamber PTW TM 31010 (Physikalisch-Technische Werkstätten (PTW), Freiburg, Germany) connected to a PTW Unidos Romeo reference-class electrometer.

The detectors were irradiated with doses ranging from 0.5 Gy to 15 Gy, and the dose rate ranged from 0.05 Gy/s to 0.26 Gy/s depending on the prescribed irradiation conditions. The irradiation time for each measurement was adjusted accordingly to deliver the required dose between 0.5 and 15 Gy. The tested dose range corresponds to individual treatment fractions in proton eye therapy (a standard ocular proton therapy course delivers a total dose of 35–70 Gy in 3–5 fractions, corresponding to fraction doses of approximately 15 Gy).

For each irradiation condition, the dose rate and irradiation (exposure) time were recorded and are provided for all measurement series in the corresponding figure/table captions. The reported timing (70 ms and 700 ms acquisition windows) refers to the integration windows used for the RL signal acquisition. The dose and irradiation conditions are summarized in Table 1.

### 3.2. FODs

Our approach, which utilizes a long optical fiber with a small-sized, well-known dosimetric or scintillation material attached as the FOD head and highly sensitive luminescence spectrometer (Figure 2b–d), offers several advantages over previously developed materials and systems for in situ dose measurements across various types of radiation therapy, especially in brachytherapy [27,28,29] and external beam therapy [30,31] (Table 1).

The Avantes AvaSpec spectrometer was selected as the readout system for the FODs. The acquisition parameters of spectrometer were optimized to maintain the detected signal within the linear dynamic range of the spectroscopic system. The detector heads consisted of GAGG:Ce, Al_2_O_3_:C and Al_2_O_3_:C,Mg scintillation crystals with typical dimensions of approximately 1.2 × 1.2 × 1 mm^3^ with a deviation of 0.05 mm, which were optically coupled to Avantes silica optical fibers with a core diameter of 1 mm and a numerical aperture (NA) of approximately 0.22. The optical fibers had a length of 7.7 m (GAGG:Ce detector) or 8.5 m (Al_2_O_3_-based detectors). A silica-based optical fiber material was selected because of its high optical transmission efficiency and good mechanical stability over the spectral range used in this study.

The optical coupling between the scintillation crystal and the optical fiber was achieved by direct mechanical contact. The assembly was fixed using medical-grade heat-shrink tubing, which ensured stable alignment of the crystal with the fiber end and reproducible light collection during the measurements. The scintillation head was positioned with its front face perpendicular to the incident proton beam axis. Only the scintillation crystal was directly exposed to the proton beam, while the optical fiber remained outside the primary irradiation field except for the short section immediately adjacent to the detector head.

The recorded RL spectra correspond to signals integrated over the selected Avantes AvaSpec spectrometer acquisition intervals (70 ms or 700 ms) during continuous proton irradiation. Each spectrum therefore represents the average RL signal collected within a single acquisition window and does not correspond to the cumulative signal over the entire irradiation period.

The first step in the measurement procedure was to record the dark spectrum without irradiating the FOD. This spectrum was subsequently subtracted from all RL spectra recorded during irradiation with doses in the 0.5–15 Gy range. In addition to dark-spectrum subtraction, we performed a crystal-free FOD-only control measurement under identical acquisition conditions. The FOD-only measurement showed only background signals and no measurable emission spectrum; in particular, no radioluminescence peak was observed. Therefore, the measured signals in the scintillator configurations can be attributed to the scintillator response.

The selected integration times were used to optimize signal acquisition for different RL intensity levels while maintaining the measurements within the linear response range of the spectroscopic detection system. No saturation effects were observed under the applied experimental conditions. Therefore, the variations in the integrated RL intensity are attributed primarily to the scintillation response of the FODs and the delivered proton dose, rather than to the integration time itself.

#### 3.2.1. FOD Based on the GAGG:Ce Crystal Head

The first detector configuration was based on a heavy GAGG:Ce crystal scintillator, which is characterized by a high density (6.5 g/cm^3^), effective atomic number Z_eff_ of 64, very high light yield (LY), fast scintillation response, and good radiation hardness, making it a suitable candidate for proton beam monitoring [18]. A schematic view of the detector assembly, including the scintillation crystal, optical fiber, and beam orientation, is shown in Figure 2.

**Figure 3:**
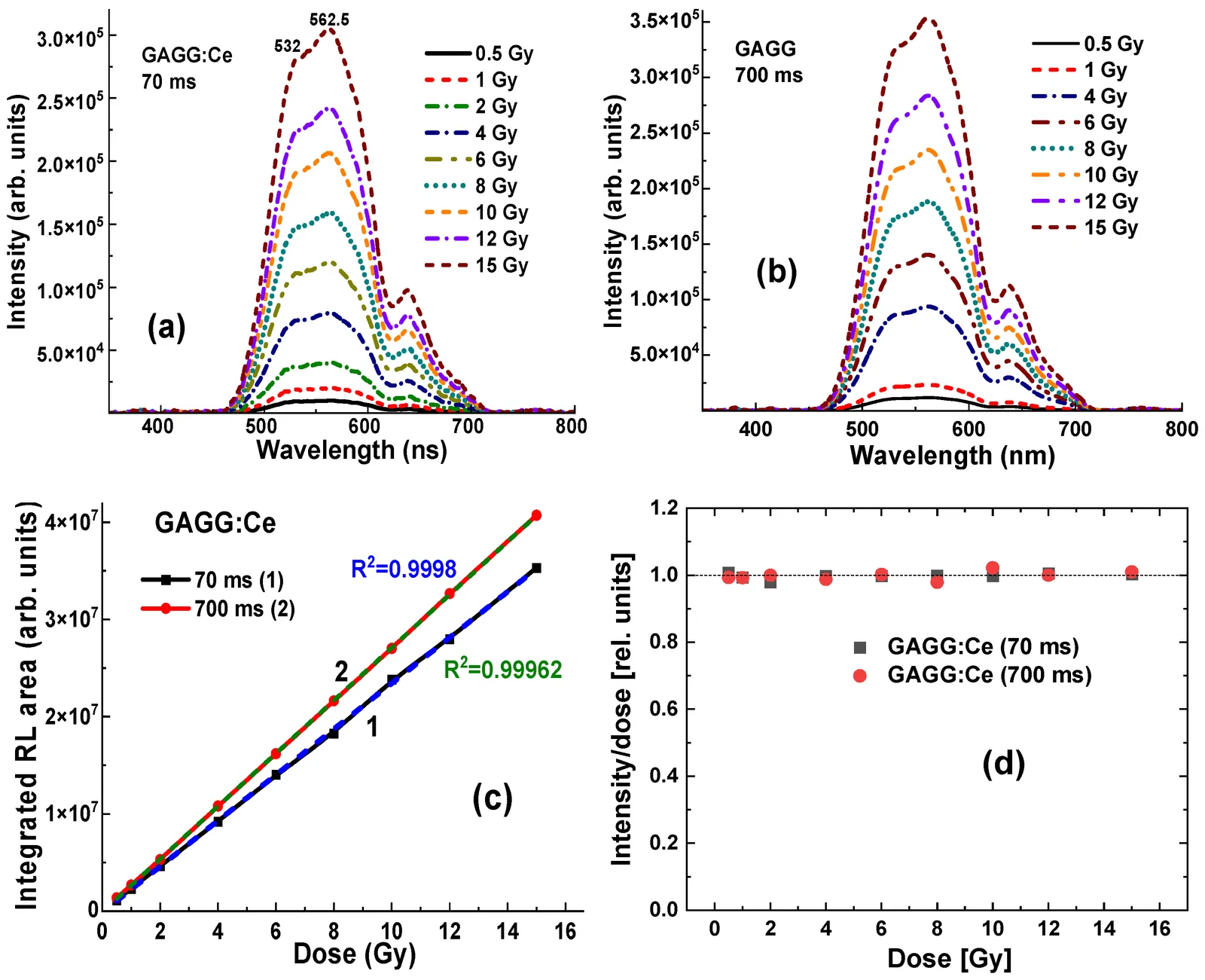
(**a**,**b**) Dependence of the RL spectral signal of the FOD based on a GAGG:Ce crystal scintillator on the proton irradiation dose in the range of 0.5–15 Gy, registered with integration times of 70 ms (**a**) and 700 ms (**b**). For each series, the dose rate and irradiation time are specified in Table 1. (**c**) Relationship between the integrated RL band area recorded with integration times of 70 ms (1) and 700 ms (2) as a function of the proton irradiation dose. The dependencies in Figure 3c are approximated by dashed lines (I = (3.4145) × 10^7^ (1) and I = (3.9414) × 10^7^ (2)) and the respective R^2^ values describe how well the approximated model fits the data. (**d**) Deviation within the measured dose–RL response function expressed as the intensity-to-dose ratio.

Figure 3a,b shows the dependencies of the RL spectra intensity on the proton radiation dose in the range of 0.5–15 Gy that were registered within time intervals of 70 ms and 700 ms, respectively. Spectra corresponding to doses between 0.5 and 15 Gy were acquired throughout the entire irradiation period. The measured parameters were the RL intensity and the integral area between the RL spectrum and the x-axis as functions of the irradiation dose. The integrated RL intensity reported in this work corresponds to individual acquisition intervals and was subsequently analyzed as a function of the delivered dose.

For both integration times, a monotonic increase in the RL intensity and the integrated RL spectral area with increasing irradiation dose was observed (Figure 3a,b), indicating stable scintillation performance under proton irradiation. The observed linear dose–response behavior within the investigated range and under the applied spectrometer integration conditions confirms the reliable performance of the investigated FODs.

A comparison of the dose–response characteristics for the two integration intervals is presented in Figure 3c. The RL band intensity demonstrates an approximately linear relationship with dose for both 70 ms and 700 ms acquisition times, with a higher signal amplitude achieved for the longer integration interval. It should be emphasized that the 70 ms values represent the spectrometer integration time rather than the intrinsic temporal resolution of the detector. These results demonstrate that stable RL spectra can be acquired with a minimum acquisition time of 70 ms using the present experimental setup. This behavior confirms the suitability of the GAGG:Ce-based FOD for real-time proton beam registration, including applications requiring fast temporal resolution.

#### 3.2.2. FODs Based on the Al_2_O_3_:C and Al_2_O_3_:C,Mg Crystal Heads

The second detector configuration employed “light” crystal scintillators based on carbon-doped aluminum oxide (Al_2_O_3_:C) and magnesium co-doped aluminum oxide (Al_2_O_3_:C,Mg). These materials with a density (3.5 g/cm^3^) and effective atomic number (Z_eff_ = 11) closer to those of tissue are widely used in luminescence dosimetry due to their high sensitivity and good dose linearity [21,22,23,24].

**Figure 4:**
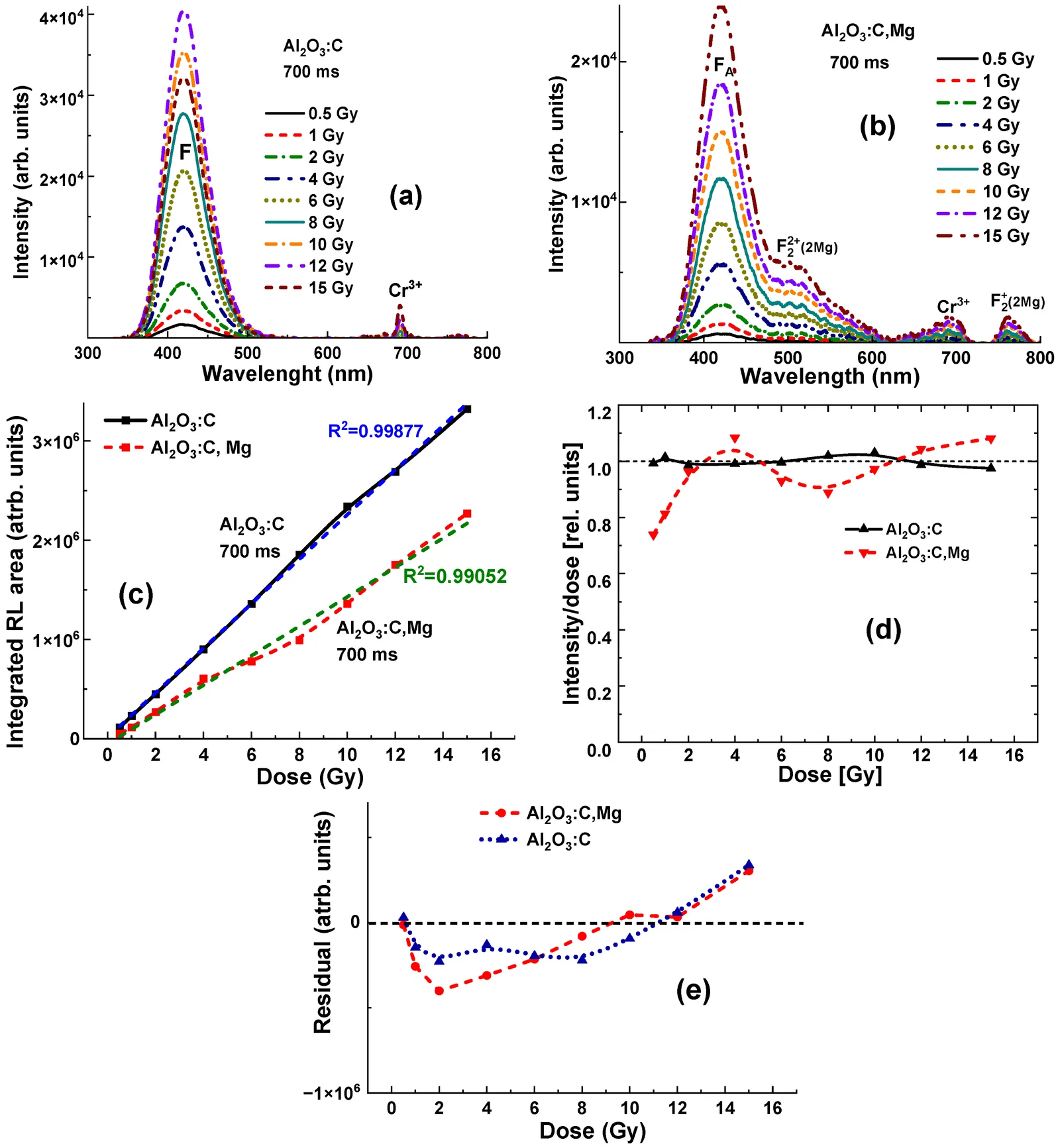
Dependence of the RL spectral signal of FODs based on Al_2_O_3_:C (**a**) and Al_2_O_3_:C,Mg (**b**) crystal scintillators on the proton irradiation dose in the range of 0.5–15 Gy. For each series, the dose rate and irradiation time are specified in Table 1. The RL signal was registered with an integration time of 700 ms. (**c**) Relationship between the integrated RL band area as a function of the proton irradiation dose. The dependencies in Figure 4c are approximated by dashed lines (I = (2.2434)10^5^ and I = (1.4816)10^5^) and R^2^ values describe how well the approximated model fits the data. (**d**) Deviation within the measured dose–RL response function expressed as the intensity-to-dose ratio. (**e**) Residual plot of “RL area vs. dose” dependencies calculated from Figure 4c for FODs based on Al_2_O_3_:C and Al_2_O_3_:C,Mg crystals.

Figure 4a,b presents the dependencies of the RL spectra on the proton radiation dose in the range of 0.5–15 Gy for FODs based on Al_2_O_3_:C and Al_2_O_3_:C,Mg, respectively. The RL spectra measurements were performed with a signal integration time of 700 ms.

Both scintillators exhibit a clear and reproducible increase in RL intensity with increasing dose (Figure 4c). The observed dose–response relationships indicate that both materials are suitable for proton beam dosimetry. Compared to the Al_2_O_3_:C detector, the Al_2_O_3_:C,Mg-based FOD demonstrates enhanced luminescence efficiency in the visible range, which can be attributed to the presence of magnesium-related trapping centers influencing the recombination processes (Figure 4b).

However, the overall RL intensity of the Al_2_O_3_:C,Mg scintillator is noticeably lower than that of its Al_2_O_3_:C counterpart (Figure 4c). This leads to a larger deviation of the signal from the generally linear trend in the measured dose–RL response function, expressed as the intensity-to-dose ratio (Figure 4c). A residual plot (Figure 4e) for the Al_2_O_3_:C and Al_2_O_3_:C,Mg scintillators shows that the residuals are generally close to zero, indicating relatively small differences between the observed and predicted values. However, a systematic pattern is evident: positive crystal scintillator residuals are at the low and high signal levels and negative residuals are in the mid-range. This suggests that, although the model provides a reasonable first-order approximation, it does not fully capture the relationship. In particular, it indicates some nonlinearity in the response of Al_2_O_3_:C,Mg and only partial fulfillment of the assumption of random, unbiased errors.

#### 3.2.3. Scintillation Response Stability and Radiation Hardness of Different FODs

**Figure 5:**
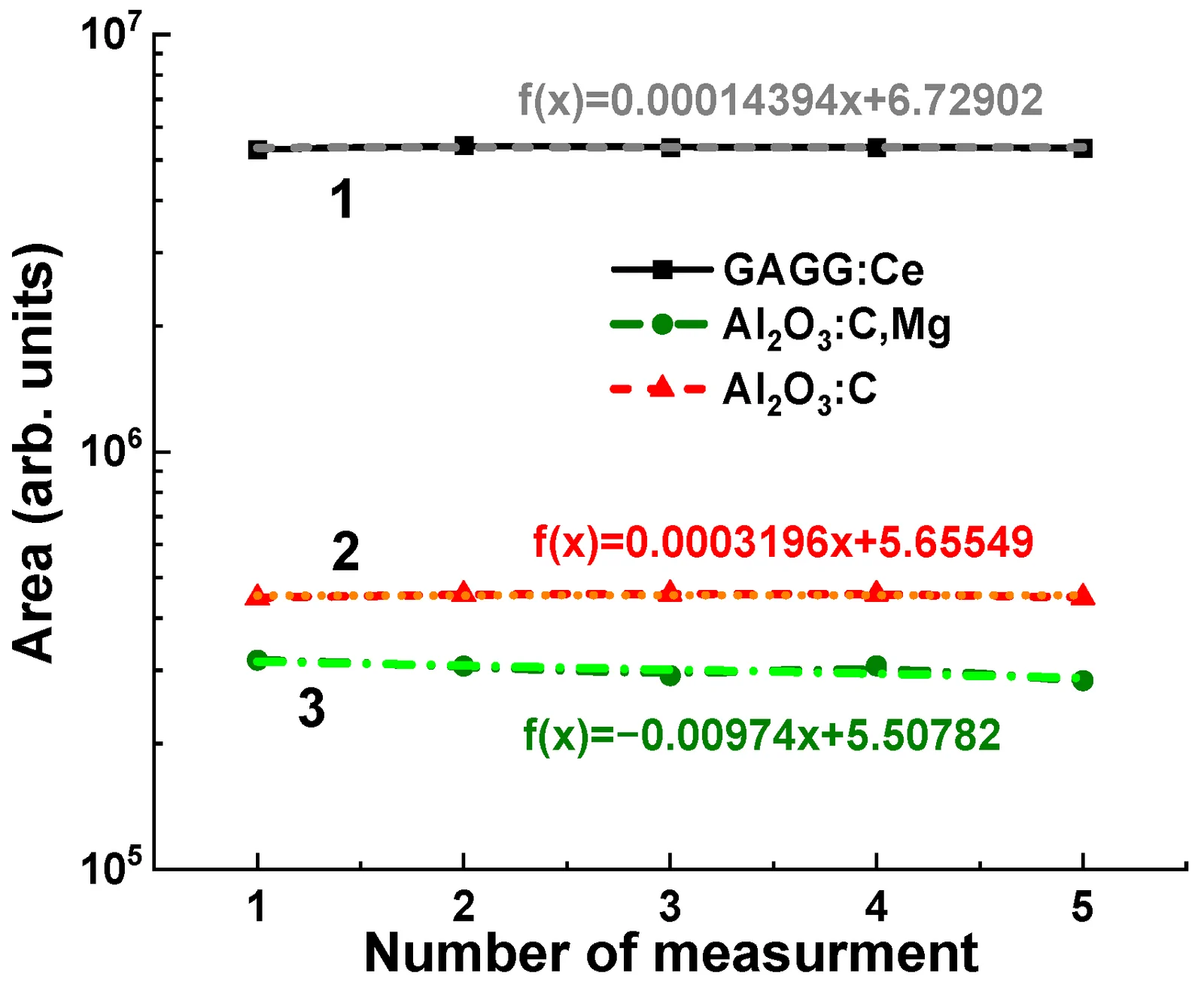
Comparative stability of the scintillation response of FODs based on GAGG:Ce (1), Al_2_O_3_:C (2), and Al_2_O_3_:C,Mg (3) crystal scintillators during a series of proton irradiation cycles with a delivered dose of 2 Gy per cycle. The dependencies in Figure 5 are approximated by dashed lines.

A comparative study of the stability of the scintillation response of FODs based on GAGG:Ce, Al_2_O_3_:C, and Al_2_O_3_:C,Mg crystals was carried out under repeated proton irradiation cycles. Each irradiation cycle delivered a dose of 2 Gy. Figure 5 illustrates the scintillation response stability of all three detector types over a series of irradiation cycles. The repeatability of the measured scintillation signals was evaluated based on the standard deviation (SD) and coefficient of variation (CV), calculated as the ratio of the SD to the mean signal value. The CV was used to compare measurement variability among the investigated materials, as it accounts for differences in mean signal intensity.

The GAGG:Ce-based FOD exhibited the highest signal stability, with minimal variation between successive cycles (CV = 0.78%; Table 1). The Al_2_O_3_:C detector also demonstrated good stability (CV = 1.09%), whereas Al_2_O_3_:C,Mg showed the largest relative variability, with a CV of 4.54%. Overall, the obtained CV values indicate good measurement repeatability for all three scintillators, with the highest reproducibility observed for GAGG:Ce, followed by Al_2_O_3_:C and Al_2_O_3_:C,Mg.

Generally, the comparative measurements demonstrate that all investigated scintillation materials are suitable for proton beam dosimetry. GAGG:Ce provides superior temporal response and stability, while Al_2_O_3_:C and Al_2_O_3_:C,Mg offer high sensitivity and good dose linearity, making them attractive for complementary dosimetric applications.

The radiation hardness of scintillation materials under high cumulative doses is an important parameter that was not systematically addressed in this work. However, the reproducibility data shown in Figure 5, obtained over multiple 2 Gy irradiation cycles, demonstrate short-term signal stability under clinically relevant dose fractionation conditions, which is a necessary criterion but not the sole criterion for radiation hardness.

Nevertheless, Al_2_O_3_:C sapphire, GAGG:Ce and other garnets exhibit very high intrinsic radiation hardness. Specifically, the formation of vacancy-type defects in these materials has only been observed under irradiation with heavy ions (Xe and Kr) at fluences exceeding 10^13^ ions/cm^2^ [33,34,35,36]. No significant optical degradation has been observed under proton irradiation up to ~5 × 10^14^ p/cm^2^ [37,38], electron irradiation up to ~3 × 10^6^ Gy [38], or irradiation with X-rays and γ-quanta at doses exceeding 10^6^ Gy [39,40,41]. Therefore, the present measurements demonstrate short-term stability under the investigated irradiation conditions. A comprehensive assessment of radiation hardness under prolonged cumulative proton irradiation is the subject of future work.

## 4. Discussion

The obtained results demonstrate that scintillation FODs based on inorganic crystals represent a reliable and versatile solution for proton beam dosimetry in the clinically relevant dose range of 0.5–15 Gy. All measurements were performed at the middle of the SOBP, corresponding to a clinically relevant region of uniform dose delivery, which makes the presented results directly applicable to patient-specific quality assurance in proton therapy. It should be emphasized that the present conclusions are restricted to 60 MeV proton irradiation at the investigated position within the SOBP. The effects of proton energy, LET variation, irradiation depth, quenching phenomena, and long-term accumulated dose were not addressed in this study and require further systematic investigation before broader clinical applicability can be established.

All investigated scintillation detector heads based on GAGG:Ce, Al_2_O_3_:C, and Al_2_O_3_:C,Mg crystals exhibited stable RL output, reproducible dose–response characteristics, and resistance to repeated proton irradiation cycles. Among the tested materials, the GAGG:Ce crystal head showed the most advantageous overall performance. Its high density, large effective atomic number, and very high light yield resulted in intense RL signals and an excellent signal-to-noise ratio. Importantly, reliable dose-dependent RL acquisition was possible with integration times as short as 70 ms, demonstrating the feasibility of real-time proton beam monitoring with acquisition times down to 70 ms. Although this acquisition rate is encouraging for rapidly varying irradiation conditions, a complete temporal characterization of the detector system is required before quantitative assessment of its applicability to pencil beam scanning.

The Al_2_O_3_:C and Al_2_O_3_:C,Mg detectors, while producing lower absolute signal intensities, demonstrated good dose linearity and high reproducibility. Their low effective atomic number (Z_eff_ ≈ 11) makes them attractive for applications where absorbed dose in tissue-like media is of primary interest. The results indicate that Mg co-doping modifies the trapping and recombination processes responsible for the luminescence of Al_2_O_3_:C. Although the contribution of visible emission centers is enhanced, the overall integrated RL signal under proton irradiation is lower than that of Al_2_O_3_:C, indicating that Mg co-doping alters the emission mechanism rather than improving the overall detector sensitivity.

The comparative irradiation cycle experiments further confirm that all three scintillation materials can maintain a stable RL output under repeated proton exposure. The slightly higher stability observed for GAGG:Ce suggests superior resistance to cumulative radiation effects, while sapphire-based detectors remain sufficiently robust for routine dosimetric applications.

Scintillation FODs combine compact geometry, electrical safety, immunity to electromagnetic interference, and real-time response, making them particularly attractive for beam diagnostics and quality assurance in proton therapy. The present results demonstrate that FODs can complement existing dosimetric systems and, in selected applications, provide unique capabilities unavailable with traditional detectors.

The demonstrated properties of scintillation FODs also indicate strong potential for several proton therapy applications, including: (i) real-time monitoring of proton beam delivery during patient-specific quality assurance; (ii) verification of beam stability and reproducibility in pencil beam scanning systems; (iii) compact beam diagnostics in experimental proton beamlines; and (iv) integration into phantoms or dedicated QA devices.

The miniature size of the detector heads and remote readout capability further facilitate deployment in environments where space constraints and radiation hardness are critical. Namely, future FOD development can focus on (i) systematic characterization of the LET-dependent response across different proton energies and depths; the development of multi-point or distributed FOD systems for spatially resolved proton dosimetry; (iii) evaluation of detector performance under ultra-high-dose-rate (FLASH-like) proton irradiation; and finally, (iv) clinical translation through long-term stability testing and standardization of calibration protocols.

Although LET-dependent quenching effects were not the focus of this study, the stable dose–response observed at the middle of the SOBP suggests that the investigated FODs are suitable for routine clinical QA. Systematic characterization of the LET dependence of the RL response for all three scintillator materials across a range of proton energies (30–200 MeV) is planned as a continuation of this work. Understanding and correcting for LET-dependent quenching will be essential before this technology can be used for accurate absolute dosimetry at arbitrary positions within different Bragg peak clinical beams; this work is planned for coming future studies.

## 5. FOD Dosimetry in Comparison with Conventional Proton Dosimetry Methods

Relative to established proton dosimetry techniques, the developed scintillation FODs offer several distinct advantages (Table 2). Ionization chambers, although serving as clinical reference standards, are bulky and unsuitable for high-resolution or in situ monitoring. Semiconductor detectors provide high sensitivity but suffer from radiation damage and energy dependence. Passive dosimeters such as radiochromic films and TLDs lack real-time readout capability (Table 2).

Despite their promising performance (Table 2), several aspects still require consideration for accurate proton beam dosimetry using scintillation FODs.

**LET dependence.** Proton beams exhibit variable linear energy transfer along their path, especially near the Bragg peak. Although stable dose–response behavior was observed in the present study, further investigations are required to quantify LET-dependent quenching effects for each scintillator material.

**Geometrical alignment and positioning.** Due to the small detector volume, precise alignment with respect to the proton beam is critical. Small positional uncertainties may influence measured signals in regions with steep dose gradients.

**Optical signal transport.** Wavelength-dependent attenuation in the optical fiber and spectrometer sensitivity can affect absolute signal intensity. These effects can be minimized through careful calibration and stable system configuration.

## 6. Conclusions

The performance of scintillation fiber-optic detectors (FODs) based on GAGG:Ce, Al_2_O_3_:C, and Al_2_O_3_:C,Mg crystal scintillators were systematically evaluated for proton beam dosimetry in the dose range of 0.5–15 Gy. We have found that all investigated detectors demonstrated stable and reproducible radioluminescence responses with a clear dose dependence.

The GAGG:Ce-based FOD exhibited the highest signal intensity, excellent stability, and reliable operation at short integration times, making it particularly suitable for real-time proton beam monitoring. The Al_2_O_3_:C- and Al_2_O_3_:C,Mg-based detectors exhibited high sensitivity and good dose linearity under the investigated proton irradiation conditions. Mg co-doping altered the luminescence mechanism by increasing the contribution of visible emission centers without enhancing the overall integrated RL response. Their relatively low effective atomic number (Z_eff_ ≈ 11) may be beneficial for proton dosimetry, although tissue equivalence was beyond the scope of the present study.

Repeated irradiation measurements were performed for each FOD configuration to assess the repeatability of the radioluminescence response. Excellent agreement between consecutive measurements confirmed the high repeatability and short-term stability of the investigated fiber-optic dosimeters under the applied proton irradiation conditions. However, these results are restricted to the experimental conditions of the present study and do not constitute a comprehensive evaluation of long-term radiation hardness.

The presented results provide practical guidance for the selection and optimization of scintillation FODs for real-time proton beam monitoring systems. They demonstrate that these detectors represent a robust, flexible, and scalable platform for proton therapy beam diagnostics and dosimetry. However, the conclusions are currently limited to measurements performed with a 60 MeV proton beam at a single position within the SOBP. Further systematic studies addressing LET dependence, proton energy variation, depth-dependent responses, quenching effects, cumulative dose behavior, and long-term radiation stability are required to fully validate the performance of these detectors and support their implementation in clinical proton therapy quality assurance and real-time beam monitoring applications.

## Figures and Tables

**Table 1:** The standard deviation (SD) and coefficient of variation (CV) characterize the repeatability of the measured scintillation signals using GAGG:Ce, Al_2_O_3_:C and Al_2_O_3_,Mg scintillators.

Scintillators	GAGG:Ce	Al_2_O_3_:C	Al_2_O_3_:C,Mg
SD	41,669	4935	13,675
Mean	5,363,593	453,391	301,263
$\mathrm{CV}=\frac{SD}{Mean}\times 100\%$	0.78%	1.09%	4.54%

**Table 2:** Comparison of materials and methods for proton beam dosimetry.

Detector Type/Method	Material Properties	Advantages	Limitations	Suitability for Proton Beam Monitoring
**Ionization Chamber**	Gas-filled, near tissue-equivalent	High accuracy, clinical standard	Bulky, limited spatial resolution, slow response	Reference dosimetry, not ideal for real-time monitoring
**Semiconductor Detector**	Si, diamond	High sensitivity, compact	Radiation damage, energy dependence	Beam profiling, limited long-term stability
**Radiochromic Film**	Polymer-based	High spatial resolution	No real-time readout, post-processing required	QA and verification
**Thermoluminescent Dosimeter (TLD)**	LiF, Al_2_O_3_	High sensitivity	No real-time signal, annealing required	Retrospective dosimetry
**FOD (GAGG:Ce)**	High density (6.5 g/cm^3^), Z_eff_ ≈ 64, high LY	Fast response, high signal intensity, excellent stability	Not tissue-equivalent	Real-time beam monitoring, fast beam delivery
**FOD (Al_2_O_3_:C)**	Density of 3.5 g/cm^3^, Z_eff_ ≈ 11, low Z	Good dose linearity, high sensitivity	Lower signal than GAGG:Ce	Clinical dosimetry, QA
**FOD (Al_2_O_3_:C,Mg)**	Modified trapping centers	Enhanced luminescence efficiency, stable response	Slightly longer integration needed	High-sensitivity proton dosimetry

## Data Availability

The original contributions presented in this study are included in the article/Supplementary Materials. Further inquiries can be directed to the corresponding authors.

## Supplementary Materials

The following supporting information can be downloaded at: https://www.mdpi.com/article/10.3390/ma19173724/s1. Table S1. Characteristic of proton beam irradiation of FODs based on the GAGG:Ce, Al_2_O_3_:C and Al_2_O_3_:C,Mg crystals.
