# Supplementary material for: Scintillation Fiber-Optic Detectors for Dosimetry of 60 MeV Proton Beam
Source: Materials (Basel). 2026 Sep 1;19(17):3724. doi: 10.3390/ma19173724 (PMC13566925; doi:10.3390/ma19173724)
Supplement: Supplementary file 1 [file materials-19-03724-s001.zip › materials-4441928-supplementary.pdf]

Supplemental material

Table S1. Characteristic of proton beam irradiation of FODs based on the GAGG:Ce, Al_2O_3:C and Al_2O_3:C,Mg crystals

Nr of irradiation	Crystals	Dose [Gy]	Irradiation time [s]	Dose rate [Gy/s]	Integration time	Remark
1	GAGG:Ce	0.51	8.5	0.060	70 µs	
2	GAGG:Ce	1.00	20.5	0.049	70 µs	
3	GAGG:Ce	1.99	31.5	0.063	70 µs	
4	GAGG:Ce	4.01	33.4	0.120	70 µs	
5	GAGG:Ce	5.98	48.6	0.123	70 µs	
6	GAGG:Ce	8.00	62.9	0.127	70 µs	
7	GAGG:Ce	10.01	43.2	0.232	70 µs	
8	GAGG:Ce	11.97	51.4	0.233	70 µs	
9	GAGG:Ce	14.97	60.2	0.249	70 µs	
10	GAGG:Ce	0.51	9.9	0.052	700 µs	
11	GAGG:Ce	1.00	18.2	0.055	700 µs	
12	GAGG:Ce	1.98	31.7	0.063	700 µs	
13	GAGG:Ce	4.00	34.7	0.115	700 µs	
14	GAGG:Ce	5.99	53.9	0.111	700 µs	
15	GAGG:Ce	8.00	65.8	0.122	700 µs	
16	GAGG:Ce	9.99	58.7	0.170	700 µs	
17	GAGG:Ce	9.99	47.7	0.209	700 µs	Repetition of 8 Gy dose
18	GAGG:Ce	11.96	47.6	0.251	700 µs	
19	GAGG:Ce	14.93	70.6	0.212	700 µs	
20	GAGG:Ce	1.98	34.1	0.058	700 µs	
21	GAGG:Ce	1.99	29.3	0.068	700 µs	
22	GAGG:Ce	1.98	32.7	0.061	700 µs	
23	GAGG:Ce	1.98	34.3	0.058	700 µs	
24	GAGG:Ce	1.99	35.7	0.056	700 µs	
25	Al_2O_3:C	14.95	61.7	0.242	700 µs	
26	Al_2O_3:C	11.97	54.8	0.218	700 µs	
27	Al_2O_3:C	9.98	39.1	0.255	700 µs	
28	Al_2O_3:C	7.98	64.1	0.124	700 µs	
29	Al_2O_3:C	5.99	50	0.120	700 µs	
30	Al_2O_3:C	4.00	31.8	0.126	700 µs	
31	Al_2O_3:C	1.99	32.3	0.062	700 µs	
32	Al_2O_3:C	1.98	36	0.055	700 µs	
33	Al_2O_3:C	1.98	34.6	0.057	700 µs	
34	Al_2O_3:C	1.99	35.5	0.056	700 µs	
35	Al_2O_3:C	1.98	33.7	0.059	700 µs	
36	Al_2O_3:C	1.01	15.5	0.065	700 µs	
37	Al_2O_3:C	0.50	9.1	0.055	700 µs	
38	Al_2O_3:C,Mg	0.51	8.3	0.061	700 µs	
39	Al_2O_3:C,Mg	1.00	17.3	0.058	700 µs	
40	Al_2O_3:C,Mg	1.99	34.3	0.058	700 µs	
41	Al_2O_3:C,Mg	1.98	34	0.058	700 µs	
42	Al_2O_3:C,Mg	1.99	32.3	0.062	700 µs	

43	Al_2O_3:C,Mg	1.99	33	0.060	700 µs	
44	Al_2O_3:C,Mg	1.98	34.1	0.058	700 µs	
45	Al_2O_3:C,Mg	4.01	37.5	0.107	700 µs	
46	Al_2O_3:C,Mg	6.00	58.7	0.102	700 µs	
47	Al_2O_3:C,Mg	7.98	77	0.104	700 µs	
48	Al_2O_3:C,Mg	7.98	71	0.112	700 µs	Repetition of 8 Gy dose
49	Al_2O_3:C,Mg	10.00	41.5	0.241	700 µs	
50	Al_2O_3:C,Mg	11.97	51.3	0.233	700 µs	
51	Al_2O_3:C,Mg	14.97	63.8	0.235	700 µs	
52	Al_2O_3:C,Mg	14.98	65.4	0.229	700 µs	Repetition of 15 Gy dose
